# GESMA: A dataset of Ghanaian environmental soundscapes for machine learning applications

**DOI:** 10.1016/j.dib.2026.112732

**Published:** 2026-03-27

**Authors:** Rose-Mary Owusuaa Mensah Gyening, Juliet Arthur, Prince Dawson Tetteh, Felix Adu Mensah Amofah, Kelvin Ackah, Enoch Acheampong, Betty Agyei Kponyo, Justice Owusu Agyemang, Emmanuel Ahene, Vida Kasore, Jerry John Kponyo

**Affiliations:** Responsible Artificial Intelligence Lab**,** Kwame Nkrumah University of Science and Technology**,** Kumasi, Ghana

**Keywords:** Environmental soundscapes, Acoustic scene analysis, African audio datasets, Sound event classification, Context-aware machine listening

## Abstract

This paper presents a dataset of real-world Ghanaian environmental soundscapes intended to support machine-listening research and sound-event classification in low-resource contexts. The collection contains 22,193 uncompressed 44.1 kHz/16-bit WAV recordings, captured using mobile devices across diverse environments, including urban spaces, educational institutions, marketplaces, transport hubs, and human non-verbal acoustic settings. Recordings were obtained under natural field conditions to retain authentic background noise, reverberation, and overlapping sound events. Each file is accompanied by structured metadata specifying category, class, subclass, location, and context, and all annotations have been manually verified to ensure label consistency and quality. The dataset addresses a critical geographic gap in global audio resources and provides culturally and acoustically representative material from Sub-Saharan Africa. It offers strong potential for applications in environmental monitoring, sound event detection, accessibility tools, hearing-assistive technologies, and broader audio-based AI systems.

Specifications Table

**Table:** 

Subject	Computer Science
Specific subject area	Artificial Intelligence, Machine Learning, Acoustic Scene, and Environmental Sound Classification
Type of data	Audio (WAV format), Metadata (CSV)
Data collection	Data were collected using 14 consumer-grade smartphones spanning a range of commonly used models, including Samsung Galaxy A12, Tecno Spark 7–10, Infinix Hot 8–12, Huawei Y7, and iPhone 7–12 Pro Max. A total of 18 field contributors used the Voice Record Pro app (Dayana Networks Ltd.) to capture audio under natural acoustic conditions. Recordings were exported as uncompressed 44.1 kHz/16-bit WAV files, with durations ranging from 7.5 to 12.4 s (mean ≈10 s). Inclusion criteria required a clear capture of the target environmental event, correct metadata annotation, and the absence of intelligible conversational speech or personally identifiable spoken content. Short public shouts, such as vendor calls or transport destination shouts, were allowed when they were the main sound being recorded and did not include any personal or identifying information. Files with a poor signal-to-noise ratio, excessive wind noise, clipped audio, or mislabelled content were excluded based on the exclusion criteria. All clips were manually screened to ensure consistent labelling and audio quality.
Data source location	• Institution: Kwame Nkrumah University of Science and Technology • City/Town/Region: Ashanti Region • Country: Ghana
Data accessibility	Repository name: Zenodo Data identification number: https://doi.org/10.5281/zenodo.18315044 Direct URL to data: https://doi.org/10.5281/zenodo.18315044
Related research article	None

## 1 Value of the Data

• This dataset enables the development of context-aware machine learning models using real-world Ghanaian soundscapes. It supports the creation of systems that generalise beyond Western-centric audio resources that dominate existing research.
• It provides culturally authentic environmental sounds, including market ambience, public infrastructure audio, and human non-verbal vocalisations, which are valuable for building and evaluating assistive and accessibility technologies such as hearing-support systems and navigation tools.
• A hierarchical labelling structure (category > class > subclass), together with detailed metadata, supports flexible research workflows ranging from broad acoustic scene classification to fine-grained sound event recognition.
• The dataset provides a benchmark for low-resource audio research and enables a fair comparison of machine listening models under naturally noisy, overlapping acoustic conditions common in Sub-Saharan urban environments.
• The recordings capture community-level acoustic signatures that can be used in environmental monitoring, noise exposure studies, socio-acoustic mapping, and public health or smart-city research.
• Data collection using consumer-grade smartphones lowers technical barriers for replication and permits researchers to extend the dataset or repeat the methodology without specialised recording hardware.

## 2 Background

Environmental soundscapes shape how individuals interact with urban and community spaces and influence the perceptions of safety, accessibility, and daily activity. Advances in machine listening have made acoustic scene understanding a key component of smart city infrastructure, environmental monitoring, and assistive technologies [1]. Most widely used environmental sound datasets, including UrbanSound8K [2] and ESC-50 [3], originate from high-income Western contexts and are recorded under controlled conditions. This narrow representation limits the capacity of sound recognition models to capture the acoustic diversity characteristic of Sub-Saharan African settings.

The presented dataset responds to this gap by documenting real-world Ghanaian soundscapes across urban centres, educational institutions, transport systems, and community environments. Data were captured in situ using mobile devices under natural conditions to preserve background noise, reverberation, and crowd variability. Each audio file was grouped under standardised categories, classes, and subclasses to ensure consistent labelling and reuse. The dataset also includes metadata such as location type, time of day, and ambient conditions to enhance contextual understanding. This information would allow researchers to model temporal and environmental variability accurately. This dataset provides a foundation for benchmarking and developing machine listening systems under diverse environmental acoustic conditions, complementing existing datasets that address similar challenges of acoustic diversity and variability [4,5].

## 3 Data Description

**Fig. 1:**
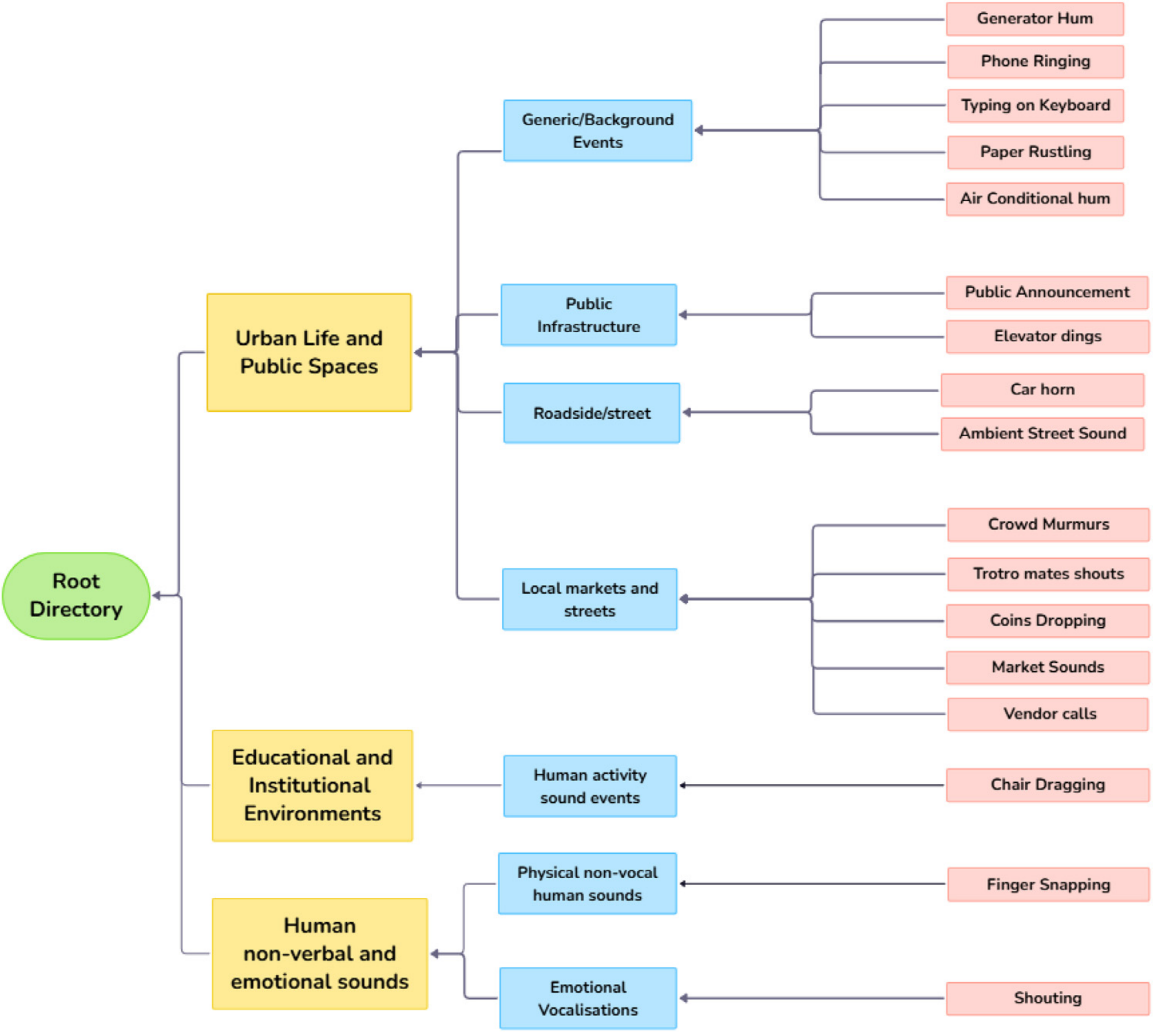
Category-class-subclass hierarchical structure of the GESMA dataset.

The dataset comprises 22,193 environmental sound recordings collected across a wide range of Ghanaian communities, including KNUST Campus, Ayeduase, Ayeduase Newsite, Boadi, Ejisu, Kejetia, Tech Junction, Adum, Atonsu, Kotei, and Gyinase, capturing a wide variety of everyday ambient acoustic environments. All recordings have been organised under a single root directory, which branches into three main categories: urban life and public spaces, educational and institutional environments, and human non-verbal and emotional sounds. Each recording file has an average duration of approximately ten (10) seconds, resulting in a combined total duration of 62.24 hours of audio data. The hierarchical directory structure supports dataset consistency, systematic management, and contextual clarity during downstream use. Fig. 1 depicts the entire dataset hierarchy, beginning at the root directory and moving through the three main categories, their classes, and finally the subclasses that represent the most detailed sound events captured.

The three main categories: Urban Life and Public Spaces, Educational and Institutional Environments, and Human Non-verbal and Emotional Sounds constitute the highest tier of organisation within the dataset repository. These categories distinguish broad acoustic contexts and provide an overarching grouping from which all subsequent levels of classification are derived. Each category aggregates recordings that share general environmental or contextual characteristics, thereby establishing a coherent foundation for the more detailed class and subclass divisions that follow. Table 1 presents a concise quantitative summary of the dataset at this top level, presenting the number of recordings assigned to each category along with their average and total durations.

**Table 1:** Category-level dataset summary.

Category	No. of Recordings	Total Duration (hrs)	Average Recording Duration(s)
Urban life and public spaces	19,110	53.69	10.11
Educational and institutional environments	1146	3.17	10
Human non-verbal and emotional sounds	1937	5.38	10

**Fig. 2:**
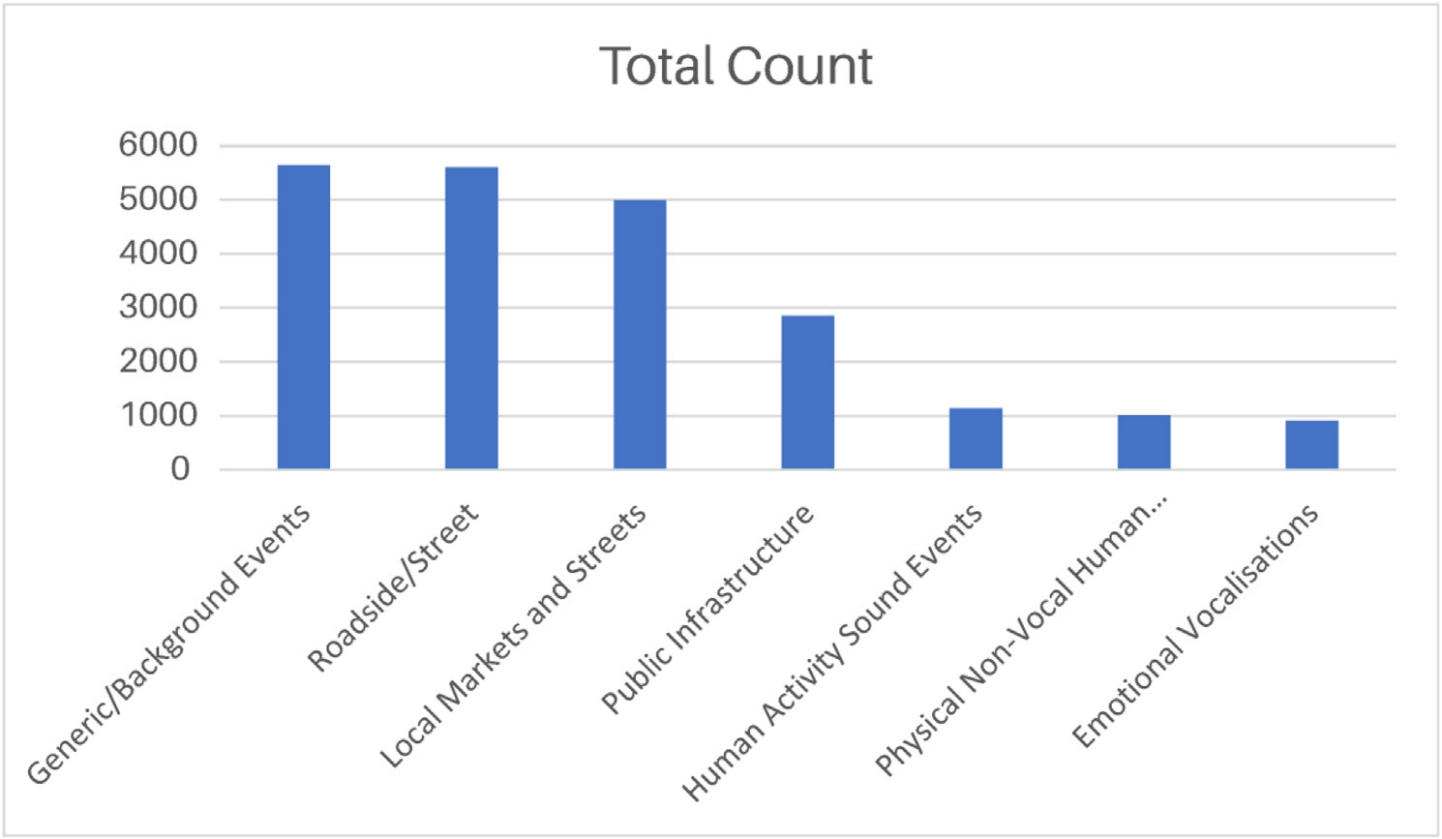
Number of recordings per class.

After categorisation into the three primary groups, the dataset is further organised into classes that provide more specific detail. This intermediate tier of organisation refines the broad thematic divisions established at the category level and defines the framework for the corresponding subclasses. Within the Urban Life and Public Spaces category, the classes include Generic/Background Events, Public Infrastructure, Roadside/Street, and Local Markets and Streets, each grouping recordings according to the nature of the activity or sound source. The Educational and Institutional Environments category contains the Human Activity Sound Events class, while the Human Non-verbal and Emotional Sounds category comprises classes such as Physical Non-vocal Human Sounds and Emotional Vocalisations. The distribution of recordings across all classes is presented in Fig. 2.

**Fig. 3:**
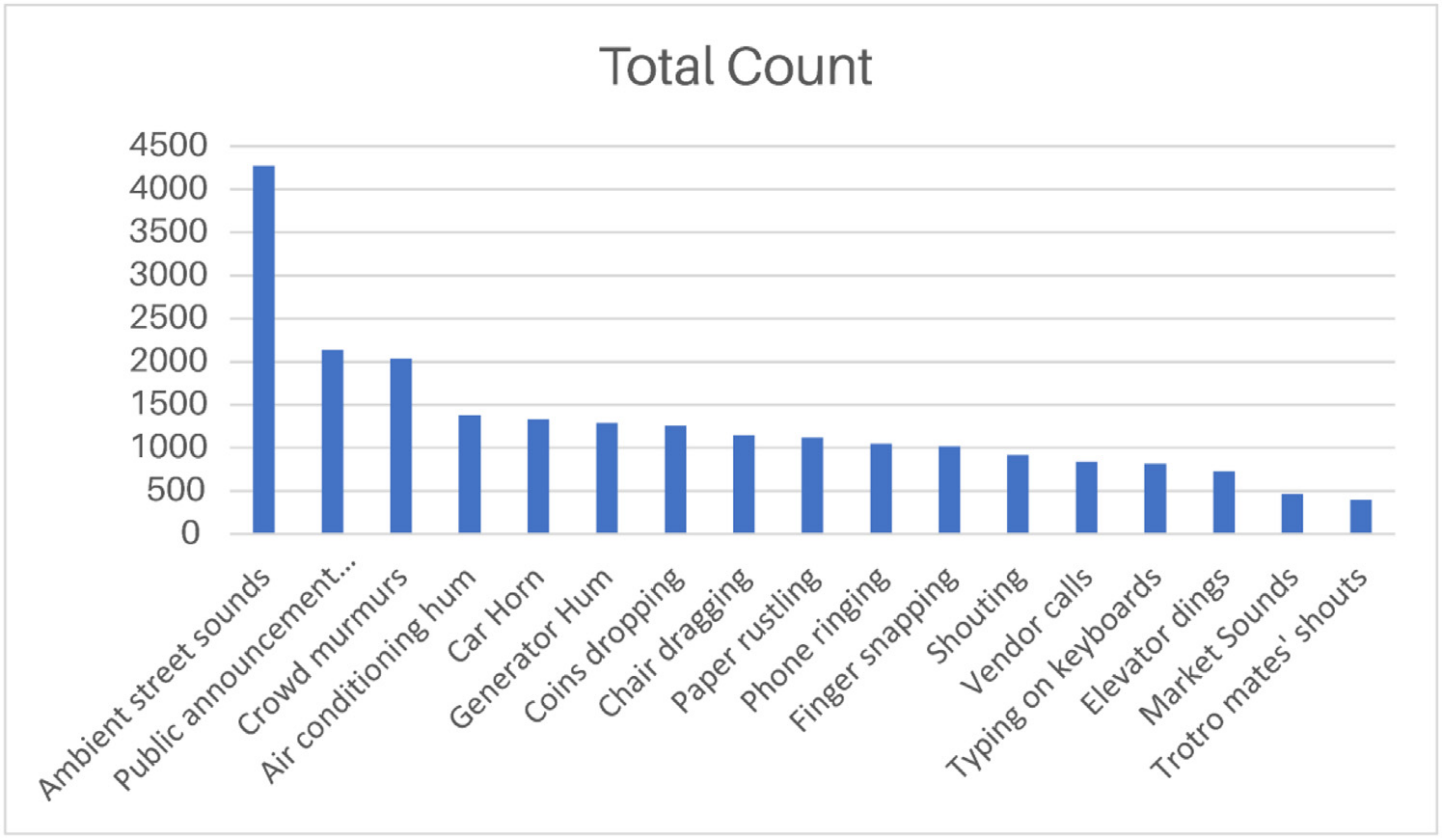
Number of recordings per sub-class.

At the most granular tier of the hierarchy, every class branches into more narrowly defined subclasses, each capturing a highly specific type of sound event in the dataset. These subclasses capture distinct acoustic phenomena such as phone ringing, typing on a keyboard, ambient street noise, crowd murmurs, shouting, and finger snapping. This level of detail enables precise differentiation between closely related sound types and preserves the diversity of auditory events recorded across the various communities. Fig. 3 illustrates the distribution of recordings across all subclasses, providing a visual representation of their relative distribution within the dataset.

**Fig. 4:**
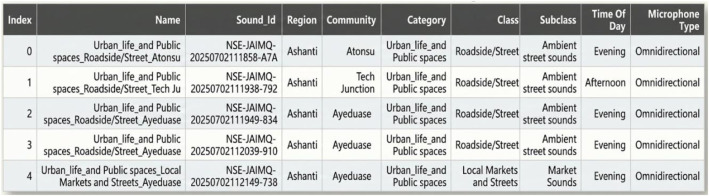
Snapshot from the CSV metadata file.

The dataset also includes a CSV metadata file containing descriptive information for each recording. Each row in the metadata corresponds to a unique audio file and is indexed using a Sound_Id that matches the audio file name. The metadata includes details about the region and community of capture, the assigned category, class, and subclass, the approximate time of day the recording was made, and the type of microphone used. This metadata file enhances the dataset’s usability by offering a structured reference that facilitates chronological sorting, traceability, and contextual analysis. Fig. 4 depicts a snapshot from the CSV metadata file.

## 4 Experimental Design, Materials and Methods

The audio dataset was developed to reflect authentic acoustic conditions found in real-world environments. Recordings were captured using commonly available smartphones, reflecting devices typically used in everyday local settings. Consistent configuration across devices ensured uniform audio quality. The Voice Record Pro application, developed by Dayana Networks Ltd., was used for all sessions, as it supports uncompressed WAV export and precise control over recording parameters. To ensure standardisation, each device was configured with identical parameters: a sample rate of 44.1 kHz, 16-bit depth, stereo channels, and high-quality Pulse Code Modulation (PCM) encoding. Environmental authenticity was prioritised by recording under naturally occurring ambient conditions, including varying crowd densities, traffic levels, and background noise. No artificial normalisation or post-processing was applied, preserving the dynamic range of real-world sounds.

The corpus comprises 17 environmental sound subclasses, human sounds, and ambient contexts. Recordings are about 10 s each, long enough to capture defining sound events without excessive repetition. Recordings were done at different times of the day to capture temporal variations in acoustic activity. Devices were handheld and positioned at approximately 0.5 to 2 m from the primary sound source, with the specific distance determined by the environmental context-closer distances (0.5–1 m) were used in relatively contained settings such as classrooms and market stalls, while greater distances (1–2 m) were maintained in open or crowded environments such as transport hubs and street settings, in order to preserve the authenticity of sound intensity and reverberation patterns.

**Fig. 5:**
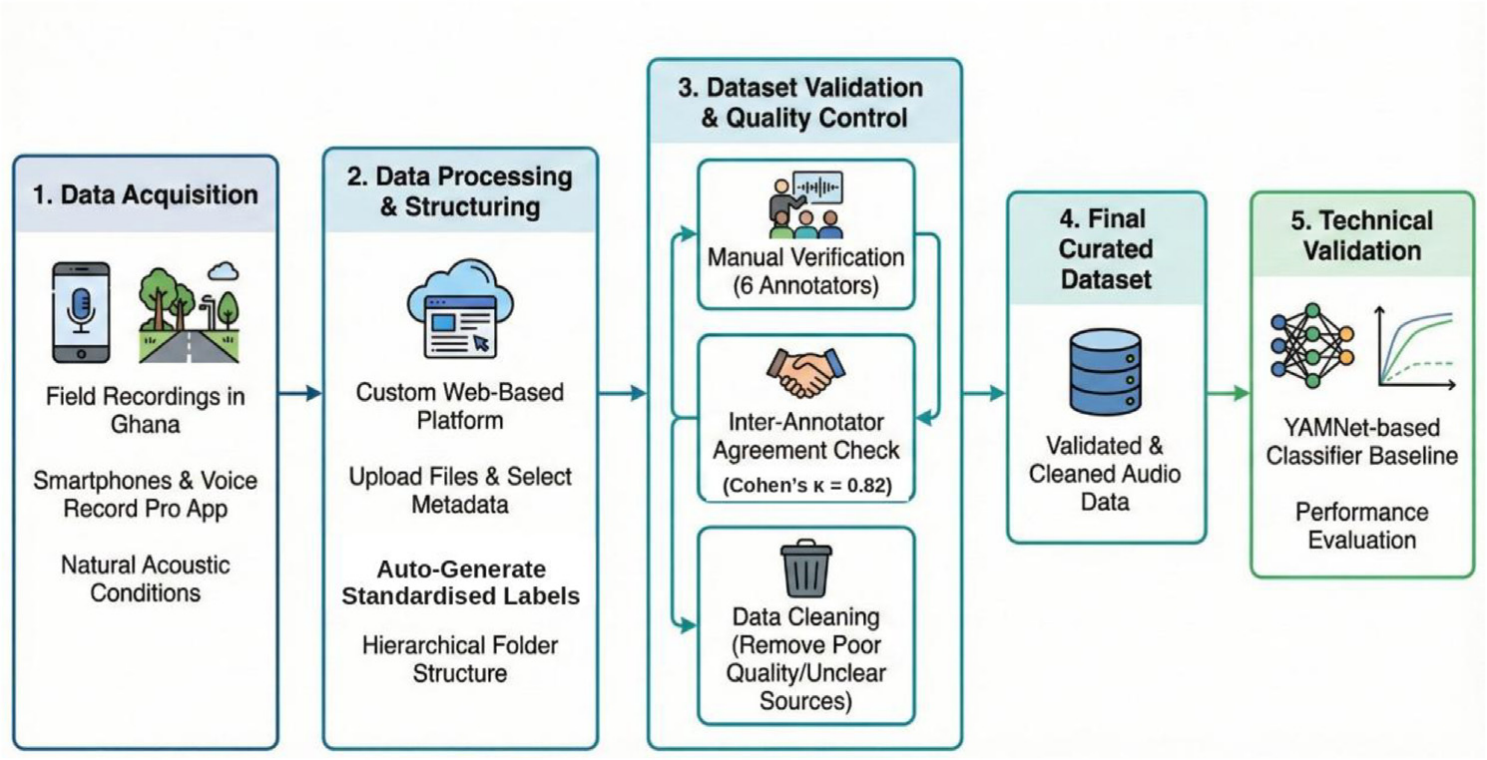
Development process for the environmental audio dataset.

A custom web-based data collection platform was developed to standardise the upload and annotation process. Contributors selected predefined metadata fields - location, category, class, subclass, and environmental context and linked them to each audio file during submission. The platform utilised these selections to automatically generate structured file labels and organise the dataset into a hierarchical folder structure, ensuring consistency and minimising manual file-handling errors. Fig. 5 illustrates the development process for the environmental audio dataset.

The dataset underwent a rigorous two-stage validation process to ensure semantic and structural reliability:

1. Manual Verification: All automatically assigned labels were verified by six (6) human annotators with experience in environmental sound identification. Each annotator reviewed a subset of the dataset to confirm label accuracy and detect qualitative inconsistencies.
2. Inter-Annotator Agreement: Inter-annotator agreement was assessed on a balanced subset of the dataset to address concerns about label reliability. Two annotators independently labelled 850 recordings (50 per subclass) while blinded to each other's annotations. Agreement was quantified using Cohen's k. The balanced subset of 50 recordings per subclass was selected to ensure statistically stable estimation of Cohen's k across all 17 categories and to reduce sampling variance in proportion-based agreement measures. This approach also prevented the majority of subclasses from disproportionately influencing the overall agreement estimate. The overall agreement was substantial (*k* = 0.82). Event-like sound categories such as chair dragging, coins dropping, finger snapping, and generator hum achieved near-perfect agreement, whereas lower agreement was observed for composite ambient subclasses such as Ambient Street Sounds, Market Sounds, and Vendor Calls, reflecting intrinsic acoustic overlap rather than annotation inconsistency.
3. Cleaning: Recordings exhibiting poor signal quality, excessive wind noise, or unclear sound sources were removed. In total, 54 files (0.24% of the collection) were excluded, leaving a final curated dataset suitable for analysis.

To demonstrate the dataset's utility for multi-class sound event classification, a baseline technical validation was conducted using YAMNet (MobileNetV1 backbone pretrained on AudioSet). The baseline evaluation was performed on the full curated dataset following the two-stage validation process, after removing 54 low-quality recordings, and not on a further filtered, cleaned subset. Audio embeddings were extracted and used to train a lightweight classifier on 17 target subclasses under a stratified train-validation-test split. Class weighting was applied during training to mitigate the substantial class imbalance inherent in real-world soundscape data. The 17 target subclasses used for this baseline are listed in Table 2.

**Table 2:** 17 target subclasses used for YAMNet-based validation.

Subclasses	Total Count
Ambient street sounds	4273
Public announcement systems	2136
Crowd murmurs	2035
Air conditioning hum	1377
Car Horn	1331
Generator Hum	1290
Coins dropping	1257
Chair dragging	1146
Paper rustling	1119
Phone ringing	1046
Finger snapping	1018
Shouting	919
Vendor calls	839
Typing on keyboards	818
Elevator dings	725
Market Sounds	466
Trotro mates’ shouts	398

The resulting model achieved an overall accuracy of 90.2%, with a weighted F1-score of 0.888 and a macro-averaged F1-score of 0.890. For context, chance-level accuracy for this 17-class task is approximately 5.88% (1/17), indicating that the baseline model performs at approximately 15 times the chance baseline, confirming that the model captures highly discriminative acoustic features. The convergence of the macro-averaged F1-score (0.890) and weighted F1-score (0.888) confirms that the class-weighting strategy effectively mitigated the influence of the dominant urban majority class, and that the reported performance reflects genuine discriminative ability across all subclasses. It is acknowledged that composite ambient subclasses such as Market Sounds, Vendor calls, and Trotro mates' shouts exhibited relatively lower performance, consistent with the annotation challenges identified during inter-annotator agreement analysis and reflecting intrinsic acoustic overlap rather than a failure of the classification framework.

While direct numerical comparison with existing benchmark datasets is constrained by fundamental differences in class taxonomy, recording conditions, and geographic context, the results reported here are contextually consistent with published YAMNet-based transfer learning performance on Western environmental sound datasets. Studies applying YAMNet-based transfer learning frameworks on UrbanSound8K - a 10-class urban sound dataset recorded under controlled conditions in North American cities - have reported classification accuracies ranging from approximately 94% to 96% [6,7]. On ESC-50, a 50-class controlled-condition dataset, comparable YAMNet-based approaches have similarly reported accuracies in the range of 94% [7]. The GESMA baseline accuracy of 90.2% across 17 classes is therefore broadly comparable to, and in several cases competitive with, these results, despite three important compounding challenges that do not apply to those datasets: (i) GESMA recordings were captured under fully uncontrolled natural field conditions with consumer-grade smartphones, introducing substantially greater acoustic variability than the studio-quality or professionally sourced recordings in UrbanSound8K and ESC-50; (ii) the class taxonomy includes culturally specific Ghanaian sound events - such as Trotro mates' shouts and Vendor calls - that have no acoustic analogues in AudioSet, the pretraining corpus of YAMNet, placing the model in a genuine out-of-distribution scenario for those classes; and (iii) the dataset exhibits significant class imbalance, with the urban majority class comprising approximately 86% of the corpus. That the model achieves a macro F1 of 0.890 under these conditions, confirms that GESMA provides a sufficiently structured and discriminative acoustic foundation for machine learning applications, while composite ambient sound classes - particularly those with strong spectral overlap - represent well-defined open problems for future research.

## Limitations

This audio dataset presents a valuable resource for acoustic research and machine listening studies; however, several limitations are acknowledged. The dataset is currently limited to recordings collected across selected locations in Ghana, which may affect its generalisability to other countries or regions with different environmental and cultural soundscapes. All recordings were captured using consumer-grade smartphone microphones, which may exhibit device-specific frequency response characteristics and dynamic range limitations. While this enhances ecological validity and replicability, models trained exclusively on this dataset may require additional calibration when applied to professional-grade acoustic sensors or specialised recording equipment. Additionally, the dataset exhibits notable class imbalance; it is dominated by recordings from Urban Life and Public Spaces (19,110 clips; ∼86% of the corpus), and learned models may implicitly rely on urban acoustic priors. To directly address the concern that the baseline model's high accuracy could be a byproduct of this imbalance, it is noted that the macro-averaged F1-score of 0.890, which weights all 17 subclasses equally regardless of their sample size, closely matches the weighted F1-score of 0.888. This convergence indicates that the class-weighting strategy applied during training successfully mitigated the dominance of the urban majority class, and that the reported accuracy reflects genuine discriminative performance across subclasses rather than an artefact of distributional skew. Nevertheless, when applied to more rural Sub-Saharan contexts, where soundscapes may contain fewer anthropogenic broadband sources and different event mixtures, performance may degrade due to domain shift. We therefore recommend (i) evaluating models on rural hold-out audio where available, (ii) using class-balanced sampling or class-weighted losses during training, and (iii) fine-tuning with a small amount of rural target-domain data (domain adaptation) if deployment is intended outside urban/peri‑urban settings.

Background noise, overlapping sound events, and slight variations in microphone distance could influence signal clarity in some samples, reflecting the challenges of uncontrolled recording conditions. Additionally, the dataset does not include detailed acoustic parameters such as sound pressure level, temperature, humidity, or perceptual attributes (e.g., urban density, occlusion, interference levels), which could be useful for advanced acoustic modelling. This absence limits the dataset's applicability for fine-grained acoustic modelling. Furthermore, although approximate recording distances are reported in the methodology, precise per-recording distance values were not logged as structured metadata, limiting future users' ability to normalise sound intensity across clips. Distance-to-source is therefore identified as an additional recommended metadata field for future extensions of this dataset, which would further strengthen context-aware machine listening applications.

## Ethics Statement

Ethical Compliance and Human Subjects Ethical approval for the dataset collection procedures were granted by the Committee on Human Research, Publication and Ethics at Kwame Nkrumah University of Science and Technology (Ref: CHRPE/AP/551/25). The research was carried out in accordance with the Declaration of Helsinki, and informed consent was obtained for all recordings involving identifiable human sound sources. For recordings conducted in public environments (e.g., markets and transport junctions), a structured privacy protocol was implemented. Recordings targeted ambient soundscapes and non-verbal acoustic events rather than conversational speech. After collection, each audio file was manually reviewed by the research team. Speech was treated as intelligible conversational content (and excluded) when a listener could reliably transcribe multi-word phrases that formed a conversation or message, including turn-taking dialogue, greetings, requests, names, phone numbers, addresses, or any content that could reasonably identify individuals. Speech was treated as non-identifying lexical call-outs (and retained) when it consisted of short, context-bound public shouts used as environmental cues (e.g., vendor advertisement words/phrases or trotro destination calls) without names, numbers, or private details, and where no conversational exchange was present. Each clip was manually reviewed; ambiguous cases were resolved conservatively by excluding the clip.

## CRediT Author Statement

**Rose-Mary Owusuaa Mensah Gyening:** Conceptualization, Methodology, Software, Formal analysis, Writing- Original draft, Supervision, Data curation, Writing- Reviewing and Editing. **Juliet Arthur:** Methodology, Software, Data curation, Validation, Investigation, Writing – Original draft, Writing – Reviewing and Editing. **Prince Dawson Tetteh:** Methodology, Software, Data curation, Validation, Visualization, Writing – Original draft, Writing – Reviewing and Editing. **Felix Amofah:** Software, Visualization, Writing – Original draft, Writing – Reviewing and Editing. **Kelvin Ackah:** Software, Visualization, Writing – Original draft, Writing – Reviewing and Editing. **Justice Owusu Agyemang:** Investigation, Validation, Writing – Reviewing and Editing. **Vida Kasore:** Investigation, Validation, Writing – Reviewing and Editing. **Betty Agyei Kponyo:** Investigation, Writing – Reviewing and Editing. **Enoch Acheampong:** Investigation, Writing – Reviewing and Editing. **Emmanuel Ahene:** Investigation, Formal analysis, Writing – Reviewing and Editing. **Jerry John Kponyo:** Conceptualization, Methodology, Resources, Supervision, Project administration, Funding acquisition, Writing – Reviewing and Editing.

## Data Availability

ZenodoGESMA: A Dataset of Ghanaian Environmental Soundscapes for Machine Learning Applications (Original data).
